# No aggregate deforestation reductions from rollout of community land titles in Indonesia yet

**DOI:** 10.1073/pnas.2100741118

**Published:** 2021-10-18

**Authors:** Sebastian Kraus, Jacqueline Liu, Nicolas Koch, Sabine Fuss

**Affiliations:** ^a^Mercator Research Institute on Global Commons and Climate Change, 10829 Berlin, Germany;; ^b^Department of Economics of Climate Change, Technical University of Berlin, 10623 Berlin, Germany;; ^c^Hertie School, 10117 Berlin, Germany;; ^d^Potsdam Institute for Climate Impact Research, 14473 Potsdam, Germany;; ^e^IZA Institute of Labor Economics, 53113 Bonn, Germany;; ^f^Institute of Geography, Humboldt University of Berlin, 12489 Berlin, Germany

**Keywords:** land tenure reform, stacked difference-in-differences, Indonesia, conservation, restoration

## Abstract

In Indonesia, 60 million people live within 1 km of state forest. The government of Indonesia plans to grant community titles for 12.7 million hectares of land to communities living in and around forests. These titles allow for using nontimber forest products, practicing agroforestry, operating tourism businesses, and selective logging in designated production zones. Here, we estimate the early effects of the program’s rollout. We use data on the delineation and introduction date of community forest titles on 2.4 million hectares of land across the country. We find that, contrary to the objective of the program, community titles aimed at conservation did not decrease deforestation; if anything, they tended to increase forest loss. In contrast, community titles in zones aimed at timber production decreased deforestation, albeit from higher baseline forest loss rates.

In 2015, Indonesia was the world’s fourth largest emitter of greenhouse gas emissions. Almost 60% of its carbon emissions were from land use change (1). Since 2016, deforestation in Indonesia has fallen by 30% (comparing 2009–2016 with 2017–2019) (2). This has, in part, been credited to a policy mix including bans on primary forest clearing and peat drainage, a review of land concessions, and a moratorium on new palm oil plantations and mines (3). Meanwhile, certification has helped protect forests on existing plantations (4). Indonesia has also initiated a large-scale land titling program that consists of the release of land for agriculture and a social forestry component. The social forestry program aims to title 12.7 million hectares as community forest. By improving livelihoods, resolving tenure conflicts, and involving communities in forest management, the program also aims at slowing deforestation. Here, we investigate the early effects of the rollout of the Indonesian social forestry policy on forest loss. We analyze data on the extent and the titling year of 4,349 land titles covering 2.4 million hectares (median size 70 ha). Our sample of titled areas runs from 2009 to 2019, and titling accelerated markedly after 2016. We compare these treated areas to control areas (median size 55 ha) from the pool of candidate areas designated for titling by the Ministry of Environment. We use satellite-based measurements of annual forest loss between 2001 and 2019 (2, 5) to compare changes in treatment and control areas before and after the introduction of each land title with a regression-based stacked difference-in-differences design.

In Indonesia, much of the land on the outer islands is designated as state forest zone. Consequently, 6 million people live on and 60 million live within 1 km of land designated as state forest (own estimates; *SI Appendix*). The population close to forest frontiers still relies, at least partially, on land for their livelihoods, either for nontimber forest products and smallholder agriculture or for ownership of or work on plantations. Devolving land rights to communities has been described as an intervention to foster the sustainable use of natural resources (6), but governments tend to use community land titles in areas with low pressure on forests. It has been unclear whether they would work at deforestation frontiers. In contrast with typical programs in other countries, the Indonesian social forestry program targets comparatively densely populated, fragmented landscapes with high pressure on forest. Many of the land titles are granted for areas on the edges of primary forest (Fig. 1).

**Fig. 1:**
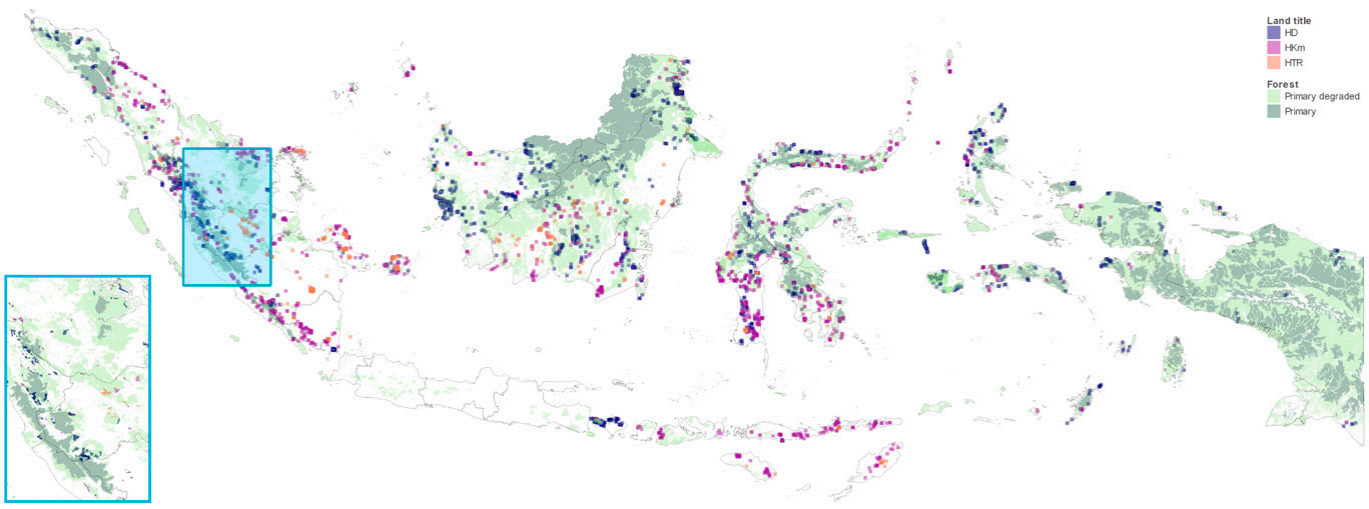
This map shows the three main types of social forestry: HD and HKm, which allow nontimber forest product collection, agroforestry, ecotourism, and some selective logging; and HTR, which aims at restoring degraded areas for community timber plantations. Primary forest and primary degraded forest in 2000 are shown in the background (5), and province boundaries are in gray. *Inset* in lower left corner is a zoomed map of the smaller area marked in blue on the main map.

The Indonesian government hopes to reduce deforestation rates by giving communities land use rights through its social forestry program (7). The program’s theory of change relates to two important observations in the literature: 1) Community institutions can mitigate the overexploitation of common-pool resources (6), and 2) smallholder practices tend to create less environmental degradation than industrial agriculture, because of locally adapted farming techniques and lower labor and transaction costs in the same household (8). In practice, the impetus for the strengthening of the social forestry program was a 2012 decision on indigenous lands by the Indonesian Constitutional Court (Court Decision 35/PUU-X/2012). Thus, the program’s principal normative goal is to correct injustices in land access. The government expects deforestation reductions only as an indirect result of reductions in land conflicts and poverty created by the policy (7). However, the success of the program critically hinges on an alignment of social forestry institutions with economic incentives and on the enforcement of community institutions in practice (overview in *SI Appendix*). Indonesian deforestation frontiers tend to be connected to markets for palm oil and other commodities with an elastic demand. This market access leads to increased land demand for plantations and shifts in the placement of other land uses, such as subsistence agriculture. High private discount rates of smallholders (9) further increase the opportunity cost of forest conservation. In addition, communities lack capacity and resources for monitoring and enforcement of social forestry institutions (10). Therefore, we hypothesize that the social forestry program cannot systematically decrease deforestation rates without additional resources or incentives.

## Results

We estimate the average countrywide effect of the community titling policy and test one of its main hypotheses: that community land titling reduces forest loss. Indonesian social forestry titles allow communities to use nontimber forest products, practice agroforestry, and operate ecotourism businesses in areas designated as protection zones by the government. In government-designated production zones, they also allow selective logging and, in specific cases (HTR titles), timber plantations.

We differentiate between the three main types of land titles: 1) village forests (*hutan desa*, HD), 2) community forests (*hutan kemasyarakatan*, HKm), and 3) community plantation forests (*hutan tanaman rakyat*, HTR). These titles are granted to villages (HD), cooperatives (HKm and HTR), or individual farmers (HTR) for 35 y (10). HD and HKm allow for restricted (50 m^3^ per year) logging for noncommercial purposes, conditional on avoiding net deforestation (11), and only in areas defined as production zones by the government. For HD titles, this is the case for 42% of the total titled area. HTR titles are established on plantation forests and degraded areas, for which communities or farmers can obtain licenses to operate and restore timber plantations. Our main sample contains 950 HD areas with a median size of 807.8 ha, 1,224 HKm areas with a median size of 246 ha, and 2,175 HTR areas with a median size of 1.3 ha.

We compare areas with land titles, before and after approval, to control areas, which are planned to get community titles in the future, in a stacked difference-in-differences design using Poisson regressions. The estimates in Table 1 can be interpreted as changes in deforestation rates in treated areas compared to control areas.

**Table 1:** Effect of community land titling on deforestation

	HD			HKm			HTR	
	(A)	(B)	(C)	(D)	(E)	(F)	(G)	(H)
	All forest	Degraded	Primary	All	Degraded	Primary	All	Degraded
Land title	0.14**	0.27***	0.35*	0.08	0.00	0.75**	–0.10	–0.83***
	[0.02, 0.26]	[0.08, 0.46]	[-0.03, 0.73]	[-0.06, 0.21]	[-0.26, 0.27]	[0.16, 1.34]	[-0.24, 0.05]	[-1.21, -0.46]
Precipitation	–0.15***	–0.16***	–0.32***	–0.15***	–0.16***	–0.31***	–0.15***	–0.16***
	[-0.20, -0.11]	[-0.23, -0.09]	[-0.52, -0.11]	[-0.20, -0.11]	[-0.23, -0.09]	[-0.51, -0.11]	[-0.20, -0.11]	[-0.22, -0.09]
Clusters	18,552	10,554	1,497	18,746	10,438	1,416	19,259	10,246
N	3,714,021	2,073,305	287,727	3,717,707	2,071,101	286,188	3,727,454	2,067,453

Estimates from Poisson regressions on a stacked sample of treatment and control groups. The row “Land title” reports the coefficient on the interaction term between an indicator for treatment and an indicator for years after treatment (*SI Appendix*, Eq. **S1**). The unit of analysis is the study area. Treated units are areas with community titles, and control units are areas designated for treatment by the government. SEs are clustered at the study area level, where treatment is assigned (see number of “Clusters”). The number of units of analysis corresponds to the number of clusters. The total number of observations (N) corresponds to all units and years in the stacked panel dataset. We differentiate between the three types of social forestry: HD, HKm, and HTR. The outcome is the deforestation rate, that is, area deforested divided by total area, at the level of the unit of observation. We show results for deforestation rates in all forest combined (2) and restricted to degraded primary forest and primary forest (5). All regressions include study area fixed effects, year fixed effects, a fixed effect indicating whether an observation is before or after the treatment year of a cohort, and a fixed effect indicating whether an observation is in the treated or in the control group for a given cohort. All regressions control for annual precipitation (CHIRPS, standardized). The 95% CI is shown in brackets. Significance levels are indicated by *, **, and *** for 10%, 5%, and 1%, respectively.

We find that the main types of community titles (HD and HKm), on average, do not decrease deforestation rates (columns A to F in Table 1). If anything, we find increases in forest loss for these two pillars of the social forestry program. However, the 95% CIs for the positive estimates, in most cases, include increases in deforestation that are small or even slightly negative. For HD titles, we can thus rule out substantial reductions in deforestation for all types of tree cover pooled into one category (column A), degraded primary forest (column B), and undisturbed primary forest (column C). This result contrasts with earlier findings based on a sample of 93 HD areas (12). Our results are stable to specifications with sample restrictions in terms of included cohorts, types of forest zones, and forest loss year observations that make our estimation sample similar to the earlier study based on a smaller sample (12). This difference could result from selection into treatment effects, because titles may be granted for the most promising areas first. It could also point to remaining omitted variable bias in the earlier study, which our stacked difference-in-difference design helps to correct (*Materials and Methods*).

An exception to the overall lack of reductions in deforestation rates can be found for HTR areas, which are granted on plantation forests. HTR areas have higher forest loss rates than HD and HKm areas before the introduction of the community title (around double for all tree cover combined, and 50% more for degraded primary forest areas). For these titles, we find substantial decreases in forest loss rates on degraded primary forest (column H in Table 1). This is indicative of increased efforts to restore forests for timber production in the HTR subsample.

## Discussion

We find that the main building blocks of the Indonesian community titling program (HD and HKm titles) overall have not led to decreases in forest loss. Case study evidence points to two main explanations for this result: 1) a lack of institutional capacity at the community level (10) and 2) the economic opportunity costs of conservation (13). Many communities lack the resources to monitor their areas or agree upon and enforce rules on resource use (10). In addition, in contexts with suitable market access for plantation-based cash crops, a reduced risk of expropriation may have led to investments in land clearing rather than restoration or forest-based activities (13). The main pillars of the program (HD and HKm) allow only the extraction of nontimber forest products and activities based on ecosystem services (for instance, tourism) or selective logging, depending on government zoning. These activities may not provide sufficient incentives to increase conservation efforts. Payments for ecosystem services, for instance, based on ecological intergovernmental transfers to villages, could help increase the value of conservation for these communities above the opportunity costs of logging and agriculture (14) or help subsistence households meet consumption needs and reduce their reliance on forests as a safety net (15).

Prior research has found forest loss reductions in a sample of early social forestry areas (12). This result indicates that, under favorable conditions, the policy can reduce deforestation. Our result indicates that, on aggregate, the program does not deliver these reductions yet.

For a subpart of the program focused on community timber production on plantations and in degraded areas (HTR titles), we find evidence for forest loss reductions, indicating an opportunity for increased conservation by including Indonesian communities in efforts to restore degraded plantations. However, HTR titles only constitute 6.3% of the granted social forestry area, and their median size is small (1.3 ha). Further research could investigate whether this result is related to increased expected future returns from standing forest due to improved tenure security or whether the reductions in forest loss merely result from less efficient harvesting, for instance, because of coordination problems between forest owners and timber companies.

## Materials and Methods

### Land Title Data

We use boundaries and land title identifiers of community titles as published by the Indonesian Ministry of Environment and Forestry (version: 14 September 2020). We also use areas designated for social forestry by the Ministry, which serve as a control group. These data are based on the Indicative Social Forestry Map PIAPS (Peta Indikatif dan Areal Perhutanan Sosial; *SI Appendix*).

### Forest Data

We use version 1.7 of the Hansen Global Forest Change data (2) and the Margono natural forest data for Indonesia (5). We reclassify the Margono categories of primary forest into primary (2, 4–9, 13–15) and primary degraded forest (1, 10–12). The outcome “all forest” is based on the Hansen data only, which detects any tree cover change, including on plantations. We use Google Earth Engine to extract the annual sum of deforested hectares for treatment and control areas (script in code repository). The outcome variable “annual deforestation rate” is the sum of deforested hectares divided by the total size of an area.

### Econometrics

We use a stacked difference-in-differences design with Poisson regressions to estimate the effect of a land title on deforestation rates. We leverage variation in the timing of treatment and use areas that have not been titled yet as a counterfactual.

The main empirical challenge is to construct credible counterfactuals for the treated areas. Governments, communities, and NGOs may work toward a title for a specific area, if it is particularly easy to lower deforestation there. However, they may also react to high local pressure on forests, leading to bias in the opposite direction. Often, the underlying factors are time variant and difficult to measure. Therefore, the direction of the bias in a research design relying on control areas matched based on measurable factors, such as topography or climate, would be theoretically unclear. We compare areas with land titles, before and after approval, to control areas that serve as counterfactual units. These counterfactual units are areas designated by the Indonesian Ministry of Environment and Forestry to get community titles in the future (PIAPS map; *SI Appendix*).

## Data Availability

Maps and code data have been deposited in Zenodo (10.5281/zenodo.4314767) (16).
